# Sub-Millisecond and Energy-Efficient Electrochemical Synaptic Transistors with a Partially Reduced Graphene Oxide Channel

**DOI:** 10.1021/acsami.5c01202

**Published:** 2025-04-19

**Authors:** Samapika Mallik, Kazuya Terabe, Tohru Tsuruoka

**Affiliations:** Research Center for Materials Nanoarchitectonics (MANA), National Institute for Materials Science, Namiki 1-1, Tsukuba 305-0044, Japan

**Keywords:** synaptic transistor, graphene oxide, Nafion, proton-gating, artificial neural network, image recognition

## Abstract

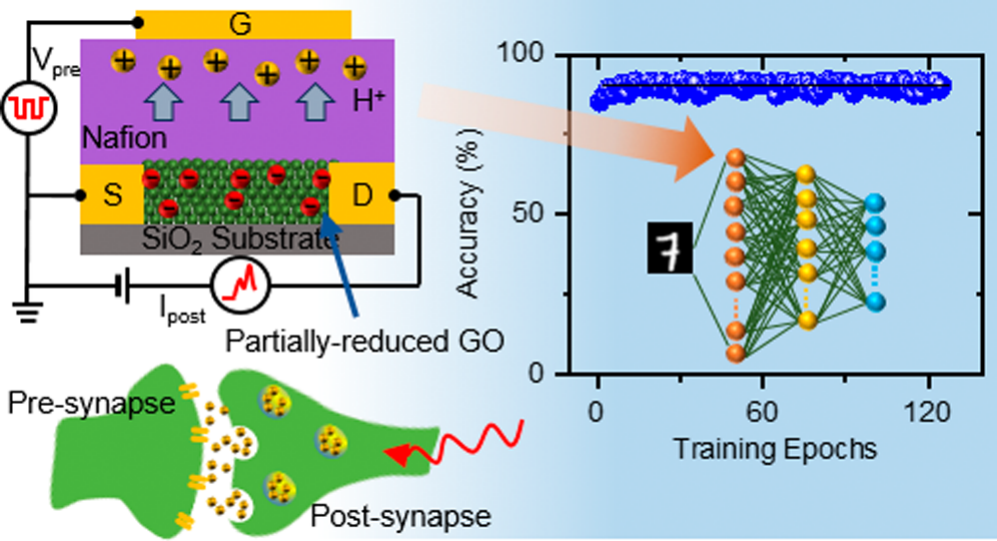

Designed artificial synaptic transistors, which emulate the functions of biological synapses, are intended to achieve information processing and computation, showcasing their promise in advancing artificial intelligence. Herein, we propose a synaptic transistor composed of a partially reduced graphene oxide (prGO) channel and a Nafion electrolyte, operating based on electrochemical reactions of the prGO channel, which are assisted by protons through the Nafion electrolyte. After electrical reduction of a pristine GO channel to the prGO channel by sweeping the drain voltage, the transistor exhibits over 200 distinct conductance states under applications of short gate voltage pulses down to 500 μs width, giving rise to a low energy consumption of 10–50 pJ per gate pulse. Using highly linear and symmetric long-term potentiation and depression characteristics, an image recognition accuracy using an artificial neural network based on a two-layer perceptron model is calculated to be 90%. If gate current pulses are used, the image recognition accuracy further increases to 94%, because of the improved linearity and symmetry of the conductance change. The transistor also exhibits short-term plasticity, such as paired-pulse facilitation and spike-timing-dependent plasticity, with time ranges of less than a few tens of milliseconds. These superior synaptic properties of the Nafion/prGO transistors will offer a remarkable paradigm for the development of neuromorphic computation architectures.

## Introduction

The human brain, an intricate computational system composed of approximately 10^11^ neurons interconnected by about 10^15^ synapses, operates with remarkable parallelism, fault tolerance, and energy efficiency.^1^ Inspired by the brain’s efficiency, neuromorphic computing systems are being developed for applications in artificial intelligence and machine learning tasks.^2^ However, the utilization of traditional graphics processing units/central processing units (CPUs) built on complementary metal-oxide-semiconductor (CMOS) circuits for simulating neural networks encounters challenges related to extensive memory requirements and elevated power consumption.^3^ This limitation arises due to the conventional von Neumann architectures of existing computer systems, necessitating substantial transfers of data between the CPU and memory.^4^ To address these challenges, neuromorphic computing systems have been proposed, characterized by their parallel processing capabilities, and offer promising avenues for analyzing vast amounts of unstructured data with enhanced efficiency in both time and energy consumption.^5−7^

Emerging artificial synaptic devices, particularly electrolyte-gated transistors, have shown promise as essential components in neuromorphic systems,^8−11^ compared to conventional CMOS-based circuits. Of the various mobile ions present in solid electrolytes, protons can offer promising benefits as smaller and more rapidly diffusing cations, leading to faster operations of such synaptic transistors. These advantages encompass reduced energy consumption, enhanced operational speed, and improved compatibility with Si technology.^12^ In the context of overcoming the scaling limitations inherent in CMOS-based synaptic devices, two-dimensional (2D) materials show promise due to their atomic-scale thickness, expansive area development, and favorable charge transport behavior. Of the possibly useful 2D materials, graphene has attracted substantial interest owing to its unique 2D planar carbon network structure and highly tunable properties.^13^ Recently, Kireev et al. reported an artificial synaptic transistor composed of a graphene monolayer channel and a Nafion electrolyte.^11^ This transistor demonstrated highly symmetric and linear conductance changes under gate current pulse applications, driven by proton movement in the Nafion electrolyte influencing graphene channel conductance. However, due to the high electronic conductivity of graphene, the conductance level ranged from several hundred to a few thousand μS, with a low on/off ratio of less than 2.

To improve the synaptic characteristics of graphene-based transistors, graphene oxide (GO) is proposed as a good alternative candidate because of its highly tunable electronic conductivity. GO is a chemically modified form of graphene, terminated with oxygen functional groups like hydroxyl, carboxyl, epoxy, and carbonyl, which render it electrically insulating.^14^ However, the redox state of GO can be controlled by applying voltage or current bias, which are otherwise known as voltage-induced or current-induced electrical reductions.^15^ prGO, a partially reduced GO, possesses both proton and electron conductivity simultaneously,^16^ indicating its potential as a mixed proton and electron conductor in electrochemical devices. The operation of a transistor based on electrochemical reactions of a GO channel was demonstrated by Tsuchiya et al.,^17^ but the synaptic behavior of a transistor with a prGO channel has not been reported to date. The partial restoration of the π-conjugated network in prGO improves its charge transport properties compared with GO, making it more suitable for mimicking synaptic behavior (e.g., gradual changes in conductance for synaptic weight adjustment). Due to the presence of residual oxygen-containing functional groups and structural imperfections, prGO can trap ions effectively, enhancing the transistor’s ability to emulate synaptic functions such as ion-modulated signal processing.

This paper reports the development of a synaptic transistor composed of a prGO channel and a Nafion electrolyte, where the channel conductance is modulated by electrochemical reactions, assisted by protons through the Nafion electrolyte in response to the gate voltage (*V*_G_). We focus on understanding the role of protons in conductance modulation to achieve superior characteristics for future physical neural networks. By utilizing the chemical patterning of a GO channel and the electron-beam lithography (EBL) patterning of a spin-coated Nafion electrolyte, the channel size can be reduced to the micrometer scale or less, which is advantageous for circuit integration. After a pristine GO channel is reduced to the prGO channel by sweeping a drain voltage (*V*_DS_), the transistor exhibits short-term and long-term synaptic plasticity under gate voltage pulses. In particular, using long-term potentiation/depression characteristics, artificial neural network (ANN) simulations based on a two-layer perceptron model exhibit high image recognition accuracy. We also discuss how the use of gate current pulses improves ANN performance as well as synaptic characteristics. To the best of our knowledge, this is the first report of an electrolyte-gated transistor using a prGO channel with good synaptic characteristics, which delivers submillisecond operation with low power consumption.

## Experimental Section

**Figure 1:**
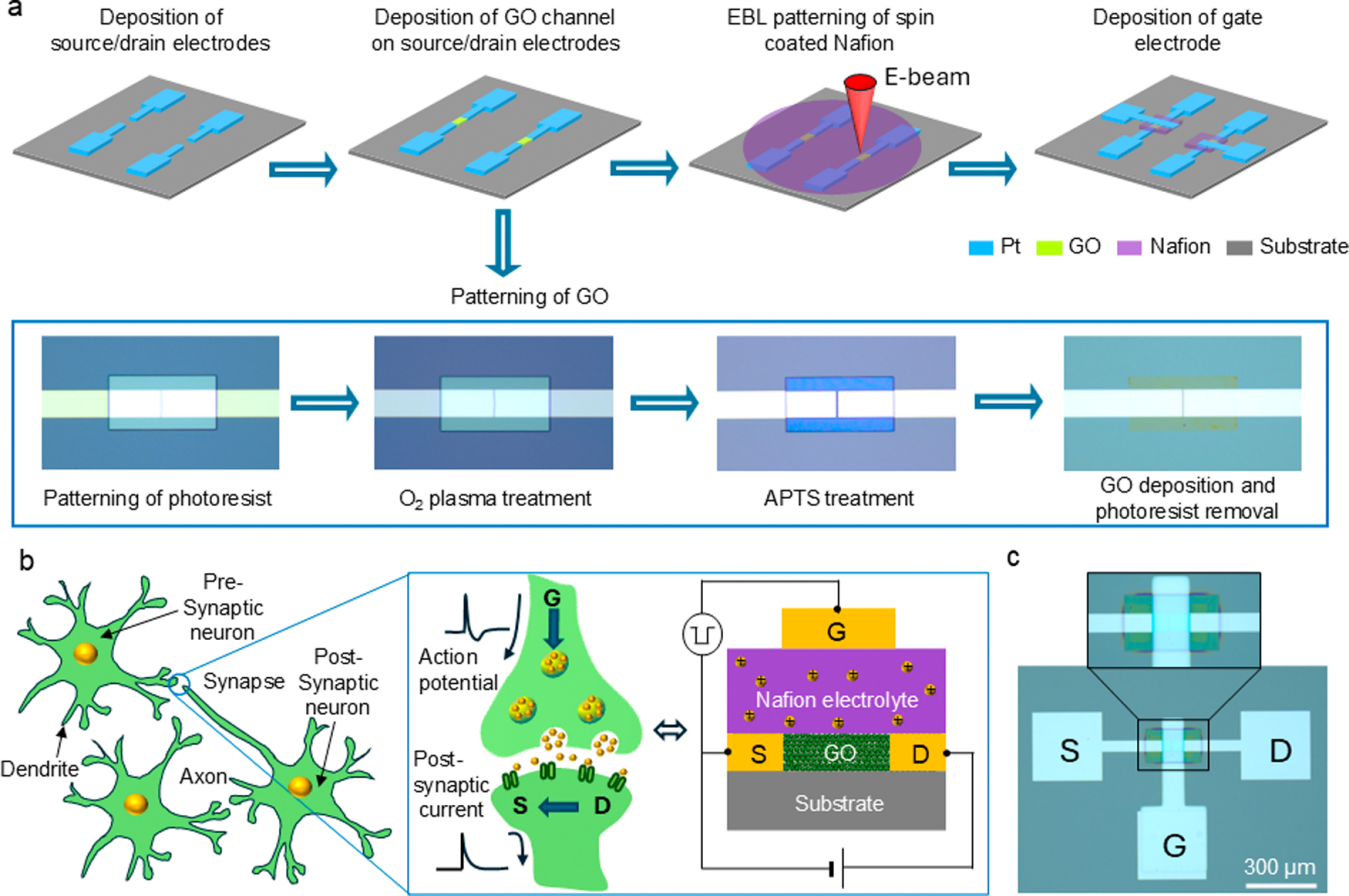
(a) Schematic of the device fabrication process, illustrating the sequential steps: deposition of source/drain electrodes, chemical patterning of GO channels, EBL patterning of spin-coated Nafion, and deposition of gate electrodes. Optical images of a GO channel with step-by-step processes show photoresist patterning, O_2_ plasma treatment, APTS modification, and GO deposition. (b) Schematic diagram of the signal transmission in a biological synapse, elucidating the analogy with our Nafion/GO transistor. (c) Optical image of the fabricated transistor.

A Nafion/GO synaptic transistor was fabricated on a Si substrate covered with a 200-nm-thick SiO_2_ layer. The transistor fabrication process is depicted in Figure 1a. First, the patterning for the source and drain electrodes, with a 2 μm length and a 50 μm-wide channel, was formed using maskless photolithography. Then, 5-nm-thick Ti and 35-nm-thick Pt were deposited as the adhesive layer and electrode, respectively, onto the patterned source and drain windows by using electron-beam (EB) evaporation. Following that, the substrate with source/drain electrodes was patterned for a channel by using photolithography. The GO used was purchased from Sigma-Aldrich. The patterning of GO flakes onto the predefined channel area (200 μm × 100 μm) involves four steps.^18^ First, the patterned substrate was treated with O_2_ plasma for 30 s to prepare a hydrophilic surface within the openings of the photoresist pattern. Then, these openings were further modified by terminating them with amine groups, inducing a positive charge under neutral conditions. This modification was achieved through immersion in a solution of 3-aminopropyl-triethoxysilane (APTS). Subsequently, the modified substrate was dipped into an aqueous dispersion containing GO flakes and then rinsed with deionized water. Finally, the photoresist was removed by a liftoff process using the remover PG, and subsequently rinsed in propanol. An optical thickness meter (Otsuka Denshi OPTM-F1) was used to determine that the thickness of the patterned GO film was approximately 5–6 nm.

After patterning the GO channel, a 200-nm-thick Nafion film was deposited using the spin-coating method. We used a 5% solution of Nafion-117 (Sigma-Aldrich) in a blend of lower aliphatic alcohols and water, and the spin-coating was executed at 3000 rpm for 30 s. The thickness of the resulting Nafion film was measured by a previously mentioned optical thickness meter. Subsequently, patterning of the coated Nafion film was accomplished by EBL,^19^ using an Elionix ELS-BODEN at an acceleration energy of 100 keV. Before commencing said patterning, the electron-beam dose of EBL was optimized to 35 μC cm^–2^ so as to preserve the integrity of the Nafion film without inducing damage or substantial changes in thickness. After the patterning, the unexposed region of the Nafion film was removed using a developer solution (a 1:1 mixture of acetone and isopropyl alcohol). Consequently, a Nafion electrolyte (dimensions: 250 μm × 150 μm) was formed above the GO channel. Finally, a 50-nm-thick layer of Pt was EB-deposited using a metal shadow mask to serve as the gate electrode with a width of 100 μm. Figure 1b illustrates an analogy between biological synapses and our synaptic transistor. An optical image of the fabricated transistor is presented in Figure 1c.

The electrical measurements of the fabricated transistors were performed under ambient conditions using a prober equipped with a semiconductor characterization system (Keithley 4200A-SCS). Throughout the measurements, the source electrode remained grounded, and the output curve was measured by sweeping *V*_DS_. The transfer curve was measured by sweeping *V*_G_ with a fixed *V*_DS_ of 0.1 V. A pulse measurement unit, connected to the Keithley 4200A-SCS, was used for voltage pulse measurements, while a source/measure unit (SMU, Keithley 2636A) was used for current pulse measurements.

## Results and Discussion

**Figure 2:**
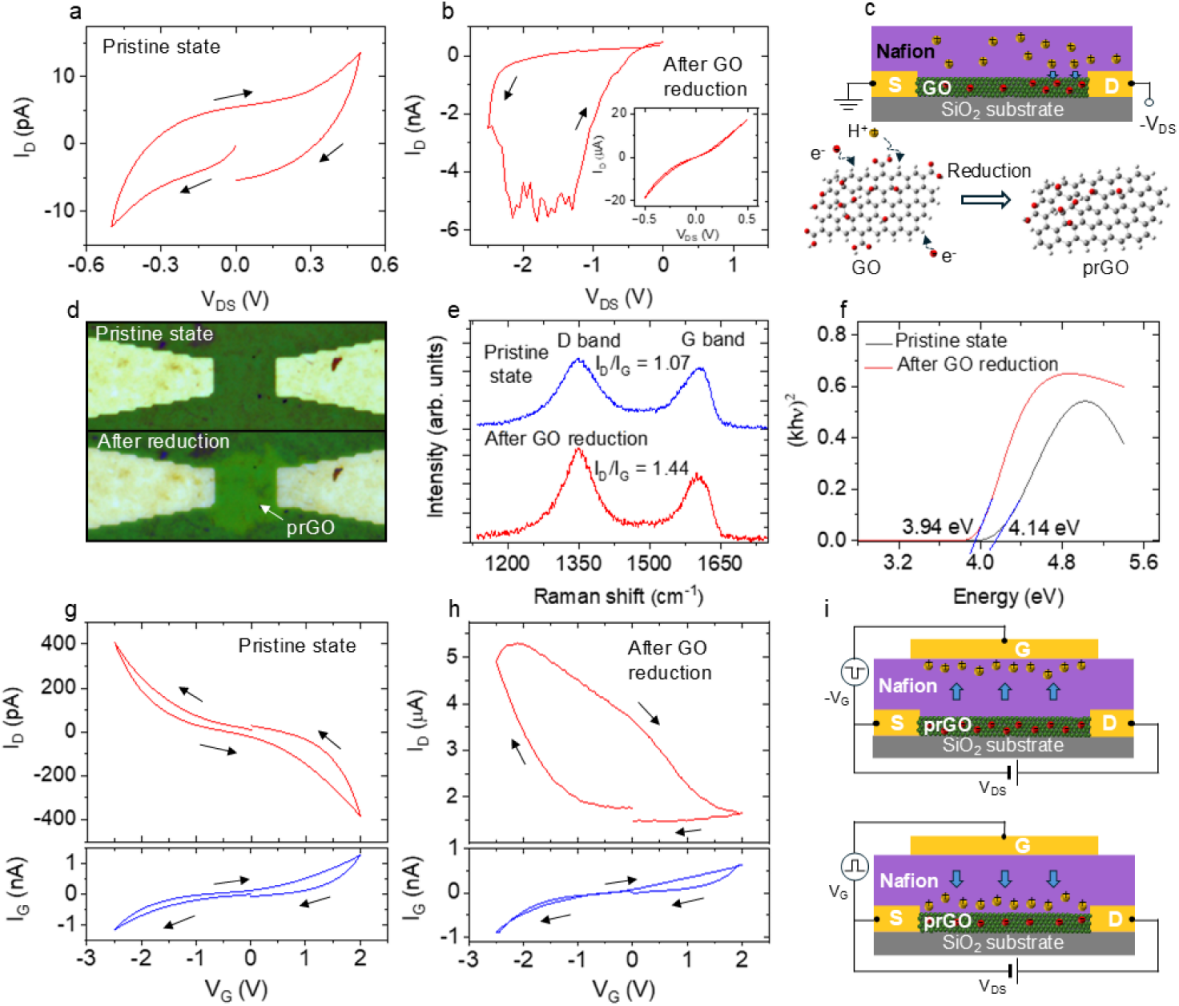
(a) Output curve of the pristine Nafion/GO transistor. (b) Variation of *I*_D_ during *V*_DS_ sweep to −2.5 V. The inset shows the output curve after repeated *V*_DS_ sweeps. (c) Schematic illustrating the electrical reduction of a GO channel to a partially reduced GO (prGO) channel under negative *V*_DS_ bias. (d) Optical images of a Nafion/GO stack formed on opposed Pt electrodes with a gap of 20 μm, taken before and after voltage sweeping to −2.5 V. (e) Raman spectra measured at the center of the GO channel before and after *V*_DS_ sweeps. (f) Tauc plots calculated by using the optical constants evaluated from reflectance measurements. The bandgap energies are determined by the intersection between the linear portion of curves and the *x*-axis. The transfer curves measured for the (g) pristine GO channel and (h) prGO channel with *V*_DS_ = 0.1 V. *V*_G_ – *I*_G_ plots obtained simultaneously with *I*_D_ are also shown. (i) Schematics of the operating mechanism for the Nafion/prGO transistor under *V*_G_ sweeps.

To study the electrical conduction characteristics of a pristine GO channel, the drain current *I*_D_ was measured under *V*_DS_ sweeps. During *V*_DS_ sweeps, the gate electrode was floating. Figure 2a shows the output curve (*I*_D_ – *V*_DS_) of the GO channel under *V*_DS_ sweep between ±0.5 V. The transistor exhibited a nonlinear current conduction of ∼10 pA, indicating a capacitive behavior of the insulating GO channel. The nonzero current at zero bias represents charge storage in the GO channel and shows the presence of states that trap electrons inside the GO layers. When the *V*_DS_ sweep was extended to ±2 V, the *I*_D_ increased to ∼1 nA, as shown in Figure S1. When *V*_DS_ was swept to −2.5 V or higher, an abrupt increase in *I*_D_ to several nA was observed, as shown in Figure 2b. After subsequently repeating *V*_DS_ sweeps several times in the same voltage range, the *I*_D_ eventually increased by 6 orders of magnitude from the initial state, and the GO channel resistance significantly decreased, leading to a linear output curve between ±0.5 V with reduced hysteresis (inset of Figure 2b). The significant increase in *I*_D_ after the negative *V*_DS_ sweep can be attributed to a partial reduction of the GO channel. The reduction of GO involves the removal of oxygen functional groups attached to its basal planes and sheet edges,^13^ which thereby restores the sp^2^ carbon–carbon bonds. This enhances electronic carrier transport by hopping between localized sp^2^ states in sp^3^ hybridizations.^13,20^ The structures of GO and reduced GO are shown in Figure S2. The redox reaction can be written as^17,21^
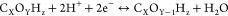
1

Various methods, including thermal reduction,^22^ chemical reduction,^21^ and electrochemical reduction,^23^ have been used to reduce GO. Reduction via voltage and current stimuli, known as electrical reduction, has also been reported.^15,24^ Yao et al. achieved the reduction of a GO film drop-cast between two Pd/Au electrodes through repeated voltage sweeps between ±2.5 V.^25^ This reduction process involves the removal of oxygen-functionalized groups according to eq 1, with the degree of said reduction being tunable by altering the voltage application conditions. The degree of reduction also depends on the device configuration and the initial nature of GO, such as its oxidation state.

In our transistor, the electrical reduction (current increase) is always initiated at a negative *V*_DS_ of −2.5 V or larger, as shown in Figure 2b. Once the reduction is initiated, further reduction occurs with both positive and negative *V*_DS_, resulting in the stoichiometric change from GO to prGO. Since the varied stoichiometry is kept under no voltage bias, the conductance state remains even after *V*_DS_ is removed, showing nonvolatile behavior. In the absence of the Nafion overlayer, no abrupt increase in *I*_D_ was observed, suggesting that the reduction of the GO channel is facilitated by the presence of protons from the Nafion electrolyte, as illustrated in Figure 2c. This aligns with eq 1 and indicates the necessity of protons for effective GO reduction. Thus, the reduction process in our transistors is closer to electrochemical reduction than that of pure electrical reduction. Quezada-Renteria et al. investigated GO film reduction in aqueous and organic electrolytes with varying proton concentrations.^26^ Protons in aqueous electrolytes were observed to promote the efficient removal of oxygen functionalities, thus enhancing GO film reduction. Their results support our experimental observation that the GO channel is reduced with protons supplied from the Nafion electrolyte under *V*_DS_ bias. Reduction of the prGO channel persisted or was further promoted even with positive *V*_DS_ bias, possibly due to the higher activation energy for oxidation or the high conductivity of prGO hindering the potential difference required for oxidation within the channel.

To examine our hypothesis of GO channel reduction by *V*_DS_ sweeps, we investigated the change in the optical properties of a Nafion/GO stack formed between two opposing Pt electrodes by micro-Raman and reflectance measurements. For these measurements, a relatively thick 41-nm GO film was patterned between two Pt electrodes with a gap distance of 20 μm, followed by EBL patterning of a spin-coated Nafion film. The optical images in Figure 2d, acquired before and after *V*_DS_ sweeps, revealed that a bright region appeared in the gap between the electrodes after the application of a −2.5 V voltage to one Pt electrode. Figure 2e depicts Raman spectra measured at the center of the GO channel before and after the *V*_DS_ sweeps. These spectra were collected using a Photon Design Jupiter with a 514.5 nm line of an Ar-ion laser and a ×50 objective lens (laser spot of ∼2 μm). To minimize heating of the sample, the laser power was kept low (0.35 mW). Both spectra consist of two main bands: a G band (∼1600 cm^–1^) and a D band (1350 cm^–1^). An increase in the relative intensity ratio of bands *I*_D_/*I*_G_ reflects a reduction of the oxidation level of the GO channel as a consequence of the large *V*_DS_ sweeping. The frequency of the G band shifted from 1605 cm^–1^ for the pristine GO channel to 1599 cm^–1^ for the *V*_DS_-biased channel, which also indicates increased portions of the reduced GO area. The additional charge carriers introduced by Nafion may change the carrier concentration in the reduced GO plane, which shifts the Fermi level toward the valence band.^27^ This decreases the band gap energy and increases the electronic conductivity of reduced GO. Micro-Raman measurements further revealed that GO is reduced from the electrode side to which a negative voltage is applied, as shown in Figure S3. Therefore, we inferred that the electrical reduction starts from the drain electrode side at negative *V*_DS_ (as illustrated in Figure 2c) and then progresses toward the source electrode side during *V*_DS_ sweeps.

Reflectance spectra were measured at the center of the GO channel by using the optical thickness meter with a light spot of ∼2 μm. The obtained spectra were analyzed with a multilayer model, in which the optical constants and the thickness of the GO layer were fitted with fixed parameters for the Nafion layer. Figure S4a plots the extinction coefficient (*k*) versus wavelength (λ), showing a red shift of about 12 nm at the onset of the *k*–λ curve after voltage bias, which indicates a reduction in the optical bandgap of the GO film. Figure S4b shows that the higher refractive index (*n*) value on the long-wavelength side also suggests a reduced bandgap energy after voltage bias. From the fitting of the reflectance spectra, the thickness of the GO film was found to decrease by 30% (from 41 to 27 nm). Voltage application induces electrolysis of water content in GO, which aids reduction by generating protons.^13^ These protons, along with those from the Nafion electrolyte, react with oxygen groups, leading to their release and a reduction in thickness. A 20–30% decrease of GO thickness has also been reported for thermally reduced GO films.^22^Figure 2f presents Tauc ((khυ)^2^ versus energy) plots calculated from the optical constants of Figure S4. The bandgap energy of the GO film was found to decrease from 4.14 to 3.94 eV after voltage bias. This supports the hypothesis of partial reduction of the GO channel by the *V*_DS_ sweep. The GO reduction was also confirmed by photoluminescence spectroscopy, as shown in Figure S5.

The transfer (*I*_D_ – *V*_G_) characteristic of the subject transistor was significantly altered by the described electrical reduction. Figure 2g,h shows the transfer curves of the pristine GO and prGO channels, respectively, measured under *V*_G_ sweeps between −2.5 and 2 V, together with *I*_G_ versus *V*_G_ plots simultaneously obtained. While the current range of *I*_G_ remained unchanged by electrical reduction, *I*_D_ increased drastically from 0.4 nA to ∼5 μA at *V*_G_ = −2.5 V. The current trajectory of *I*_D_ was symmetric for the GO channel but was considerably asymmetric for the prGO channel with a more pronounced hysteresis loop. Figure 2i illustrates the operating mechanism of the prGO channel transistor under negative and positive *V*_G_, which we propose. Native (positive) *V*_G_ causes protons within Nafion to move away from (toward) the Nafion/prGO interface. This proton migration can induce an electric double layer at the Pt/Nafion and Nafion/prGO interfaces and cause electrochemical reactions of prGO, which affect the intrinsic carrier concentration and the electronic carrier mobility, depending on the sp^2^/sp^3^ fraction in the channel. Thus, *I*_D_ is varied continuously by sweeping *V*_G_. A similar operating mechanism was proposed by Kireev et al., who attributed the modulation in I_D_ of a Nafion/graphene transistor to the migration of proton-concentrated clusters within Nafion in response to negative and positive gate current pulses.^11^ The accumulation and depletion of protons near the graphene interface shift the Fermi energy, resulting in decreased and increased conductance, respectively. Tsuchiya et al. also observed the modulation in *I*_D_ of an yttria-stabilized zirconia (YSZ)/GO transistor,^17^ which was attributed to the band gap variation arising from redox reactions of the GO channel, caused by protons provided from the YSZ electrolyte under *V*_G_ sweep above a threshold. Further investigation is needed to clarify the detailed operating mechanism of our Nafion/prGO transistor.

**Figure 3:**
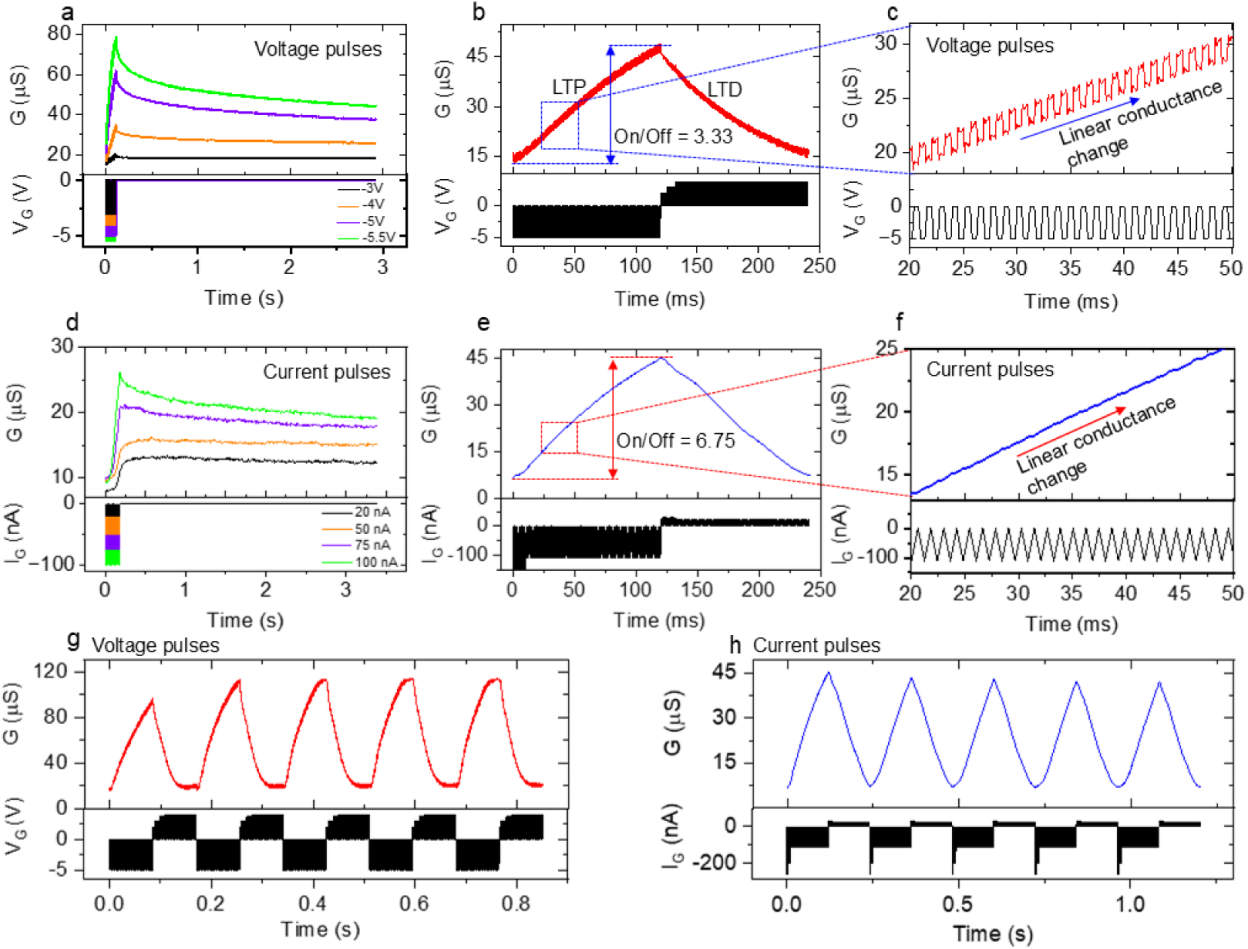
(a) Retention property of a Nafion/prGO transistor for 15 *V*_G_ pulses with different pulse amplitudes. (b) Typical LTP and LTD characteristics under application of 100 consecutive +*V*_G_ and −*V*_G_ pulse trains. (c) Magnified view of the conductance change extracted from (b). (d) Retention property for 15 *I*_G_ pulses with different pulse amplitudes. (e) Typical LTP and LTD characteristics under applications of 100 + *I*_G_ and −*I*_G_ pulse trains. (f) Magnified view of the conductance change extracted from (e). (g) Endurance cycles under 50 consecutive −*V*_G_ and + *V*_G_ pulses, with a pulse width and interval time of 1 ms. (h) Endurance cycles under 100 consecutive −*I*_G_ and +*I*_G_ pulses, with a pulse width and interval time of 500 μs.

The temporal responses to input voltage pulses are crucial when emulating the intricate plasticity mechanisms present in biological synapses. In synaptic transistors, it is essential to obtain discrete and multilevel memory states for applications in analog computation. Figure 3a shows the conductance change of the subject transistor under 15 −*V*_G_ pulses of 5 ms width and 1 ms interval time at different amplitudes (−3, −4, −5, and −5.5 V). The channel conductance, represented as “*G*”, is calculated by dividing *I*_D_ by *V*_DS_. As −*V*_G_ pulses were applied successively, the transistor showed a gradual increase in *G*, with temporary increases under pulses. With smaller pulse amplitudes (−3 and −4 V), *G* was maintained for up to 3 s after pulse applications. For amplitudes of −5 V or higher, the transistor exhibited an initial decay in *G* within the first 100 ms, followed by a gradual decrease over time and stabilization after 3 s. Using this memory characteristic, long-term potentiation (LTP) and depression (LTD) were demonstrated with consecutive −*V*_G_ and +*V*_G_ pulse trains. Figure 3b shows typical LTP and LTD behaviors, obtained with 100 voltage pulses of −5 V for LTP and 3.7 V for LTD, respectively, with the width and interval time reduced to 500 μs. To ensure linearity and symmetry in the conductance change, the pulse amplitudes were set smaller for the first ten pulses in LTD, as shown in the lower panel of Figure 3b. Under −*V*_G_ pulses, *G* increased linearly from 13.5 to 46 μS, and then returned to the initial state under subsequent +*V*_G_ pulses, resulting in an on/off ratio of 3.41. Figure 3c zooms into conductance changes during LTP, indicating a stepped increase in *G*. Distinct LTP/LTD characteristics could be observed over 200 consecutive −*V*_G_ and +*V*_G_ pulse trains in voltage pulse operations, as shown in Figure S6.

Previous works on electrolyte-gated synaptic transistors mainly utilized voltage pulse operation.^10,19^ However, large fluctuations in *G* were observed in the LTP/LTD characteristics, as shown in Figure 3b. To minimize these conductance fluctuations, current pulse operation is desired, since such operations would ensure a deterministic conductance change by injecting and extracting a constant amount of charge.^9^ Moreover, the method of electrical grounding of the gate electrode during the interval of time between applied pulses seems to be more important. In the voltage pulse operation, the gate electrode is set to 0 V, which is directly grounded. In current pulse operation, the gate electrode is set to 0 A, which is grounded through the input impedance of the SMU used. Therefore, in voltage pulse operation, discharging through the Nafion electrolyte occurs, and the channel current decays in the interval time. In contrast, discharging is significantly reduced in current pulse operation due to the high input impedance of the SMU (10^14^ Ω). This reduction in discharging improves LTP/LTD characteristics, including retention and linearity, and the on/off ratio of the channel conductance.

Figure 3d shows the conductance variation of the subject transistor under the application of 15 gate current (*I*_G_) pulses of 5 ms width and 1 ms interval times with different pulse amplitudes. The *G* value increased with increasing pulse amplitudes, which is similar to the behavior observed with the voltage pulses in Figure 3a. However, the initial conductance decay after −*I*_G_ pulses became smaller than that after −*V*_G_ pulses, in turn suppressing the decrease in G and enhancing the retention property. Figure 3e represents LTP and LTD characteristics under 100 consecutive −*I*_G_ and +*I*_G_ pulses (−110 and 25 nA, respectively), with a width and interval of 500 μs. To ensure linearity and symmetry in conductance change, the pulse amplitudes were set slightly larger for the first ten pulses in both LTP and LTD, as shown in the lower panel of Figure 3e. It was found that current pulse operation enhances the linearity of LTD compared to voltage pulse operation. The on/off ratio increased to 6.75, owing to the improved retention characteristics of current pulse operation. Figure 3f shows that *G* did not fall after each *I*_G_ pulse. Note that the channel conductance decreased by only a few percent after 50 ms with −110 nA current pulses, as shown in Figure S7. This time range is much longer than the interval time (500 μs) shown in Figure 3f. Therefore, almost no conductance decay was observed in the LTP/LTD characteristics for the current pulse operation.

Figure 3g shows five-cycle repetitions of LTP and LTD under 50 consecutive −*V*_G_ and +*V*_G_ pulse trains (−5 and 4 V), with a 1 ms width and interval time. Although the conductance change was slightly smaller in the first cycle, it stabilized after the second cycle. A similar repetition under 100 consecutive −*I*_G_ and +*I*_G_ pulse trains (−110 and 25 nA), with a 500 μs width and interval time, is shown in Figure 3h. In contrast to the voltage pulses, the conductance change was slightly larger in the first cycle but also stabilized after the second cycle. Overall, the linearity and symmetry of LTP and LTD characteristics are much better for current pulse operation than for voltage pulse operation.

We also evaluated the retention property on a longer time scale in both voltage and current pulse operations, as shown in Figure S8. In voltage pulse operation, the channel conductance dropped initially after *V*_G_ pulses and decayed rapidly to the initial state for up to 200 s. On the other hand, in current pulse operation, the channel conductance also dropped initially after *I*_G_ pulses but then decayed slowly over 1000 s. The relaxation time, defined as the time that normalized conductance decreases below 1/e, was estimated to be 37.5 and ∼600 s for voltage and current pulse operations, respectively. This result indicates that current pulses significantly improve the retention characteristics of the Nafion/prGO transistor.

It was found that environmental conditions have some effect on the performance of the Nafion/prGO transistor. Higher humidity levels resulted in larger changes in channel conductance, giving rise to larger on/off ratios of LTP/LTD characteristics, as shown in Figure S9. This variation is attributed to the increased concentration and conductivity of protons in the Nafion electrolyte at higher humidity levels. On the other hand, under vacuum conditions, the conductance change became smaller due to the desorption of water molecules from the Nafion, resulting in reduced proton conductivity. Finally, when the temperature was increased, the change in the channel conductance increased. This increased conductance arises from the increased proton conductivity of the Nafion electrolyte at elevated temperatures. To see stable characteristics of the subject transistor, all electrical measurements were performed in air, with a fixed humidity level (∼45%) at room temperature.

**Figure 4:**
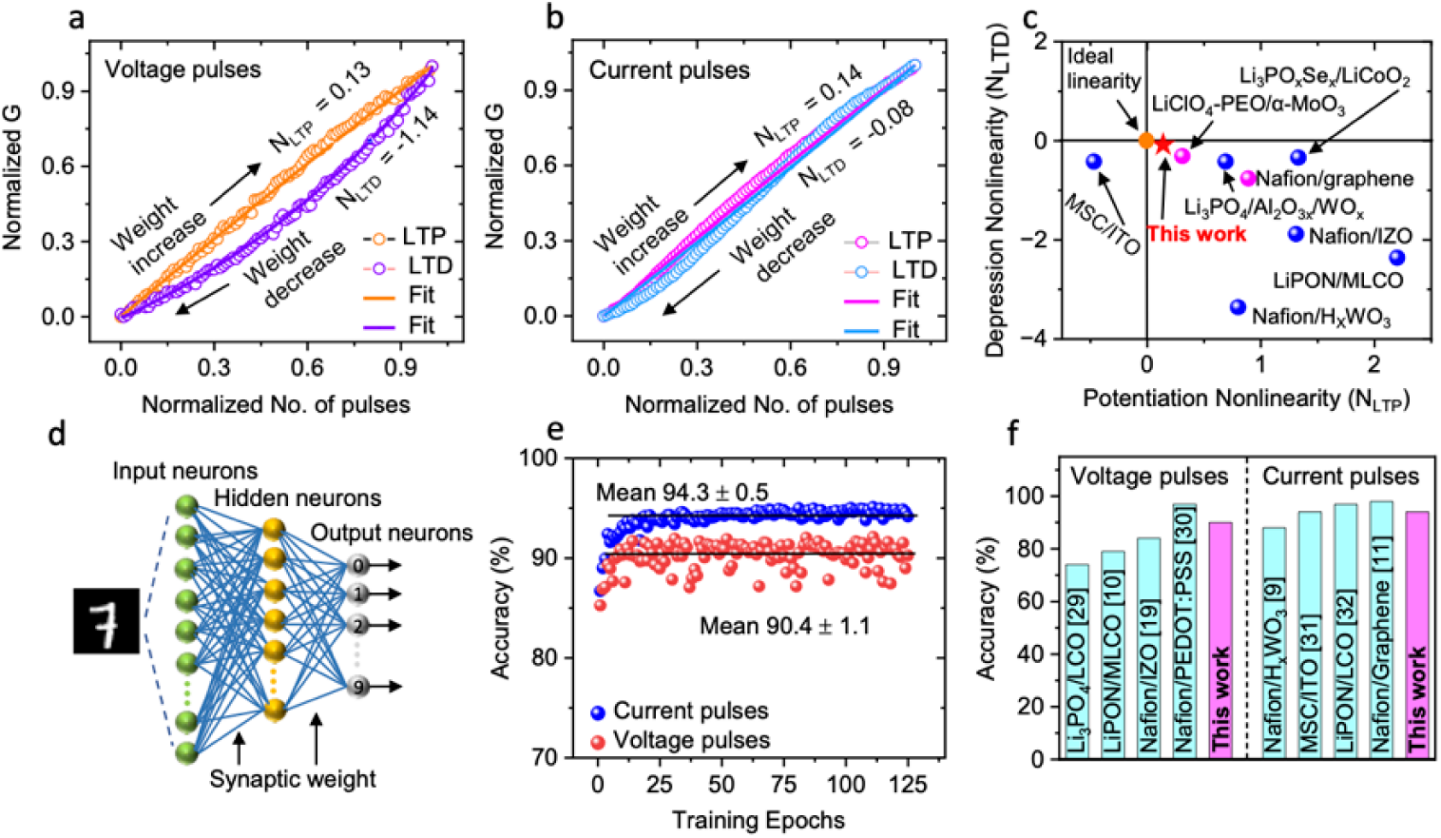
Normalized conductance versus the normalized number of pulses, obtained for Nafion/prGO transistors with (a) voltage and (b) current pulse operations. (c) Comparisons of *N*_LTD_ – *N*_LTP_ plots between the subject transistor and other synaptic transistors. (d) Schematic representation of an ANN based on a two-layer perceptron model, simulated for the recognition of handwritten digits (20 × 20 pixels) from the MNIST data set. (e) Recognition accuracy plotted as a function of training epochs, calculated for voltage and current pulse operations. (f) Comparison of recognition accuracy between the subject transistor and other synaptic transistors.

Linear conductance changes in response to input pulses are important for realizing hardware neural networks based on analog conductance-change synapses. However, actual devices generally exhibit asymmetry and nonlinearity in conductance change, which are considered to be a major obstacles for neuromorphic applications. To investigate these characteristics in the Nafion/prGO transistor, we analyzed the results of the LTP and LTD characteristics obtained in both voltage and current pulse operations. Figure 4a,b plots the normalized conductance change versus the normalized number of voltage and current pulses, respectively. These normalized conductance data were fitted with the equation of weight update, which is given as^28^
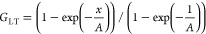
2where *G*_LT_ is the normalized conductance in LTP and LTD, *x* is the normalized number of pulses ranging from 0 to 1. *A* determines the nonlinear weight update characteristics, being positive for LTP and negative for LTD, respectively. The *A* values for LTP and LTD were determined from fitting the experimental data with eq 2 (the solid curves in Figure 4a,b). Then, the nonlinear factor for potentiation (*N*_LTP_) and depression (*N*_LTD_) was obtained from the inverse of *A* as 0.13 and −1.14 in voltage pulse operation, respectively. In current pulse operation, *N*_LTP_ was almost similar (0.14), while *N*_LTD_ was significantly improved at −0.08. A comparison of *N*_LTP_ and *N*_LTD_, with values reported for other electrolyte-gated and 2D channel-based transistors, is presented in Figure 4c. The subject transistor exhibits higher linearity of conductance change in response to current pulses compared to other synaptic transistors. The *N*_LTP_ and *N*_LTD_ values of our fabricated transistor are close to those of an ideal device.

Using the experimentally obtained results, we conducted ANN simulations for the image recognition of handwritten digits sourced from the Modified National Institute of Standards and Technology (MNIST) data set. A fully connected ANN based on a two-layer perceptron model (400 neurons in the input layer, 100 in the hidden layer, and 10 in the output layer was used for the simulations, as illustrated in Figure 4d. 20 × 20-pixel MNIST images were input, and digit classification (0–9) was performed using the NeuroSim+ platform.^28^ For the neural network training, 8000 patterns randomly chosen from the 60,000-image training data set were used. Then, inference was carried out using a separate test data set, comprising 10,000 patterns, to calculate the accuracy of image recognition at each epoch. Cycle-to-cycle and device-to-device variations (0.45% and 0.05 for the voltage pulse operation and 0.25% and 0.009 for the current pulse operation), estimated from the LTP/LTD characteristics and nonlinearity factor standard deviations, were taken into account, but these variation values were found to have almost no effect on the simulation results. The influence of device-to-device variation on the simulation results is described in more detail in Figure S10 and Table S1. Figure 4e plots the image recognition accuracy versus epochs for voltage (red spheres) and current (blue spheres) pulse operations. With voltage pulse operation, our transistor achieved a 90% recognition accuracy after 25 epochs. The recognition accuracy further increased to 94% with current pulse operation, which is the same level as an ideal device.^28^ Notably, the current pulse operation significantly reduced fluctuations in accuracy, which is due to the improved linearity and asymmetry of the conductance change. Figure 4f shows a comparison of the image recognition accuracy for various synaptic transistors with voltage and current pulse operations. The recognition accuracy of our Nafion/prGO transistor with voltage pulse operation is higher than that of Nafion/H_*x*_WO_*x*_,^9^ Nafion/IZO,^19^ and Li_3_PO_4_/WO_*x*_^29^ transistors, is comparable to that of a Li_3_PO_*x*_Se_*y*_/LiCoO_2_ transistor,^30^ and is slightly lower than that of a Nafion/PEDOT:PSS/PEI transistor.^31^ In current pulse operation, the accuracy of our transistor is higher than that of a Nafion/H_*x*_WO_3_ transistor,^9^ is comparable to that of an MSC/ITO transistor,^32^ and is slightly lower than that of Nafion/graphene^11^ and LiPON/LiCoO_2_^33^ transistors.

**Figure 5:**
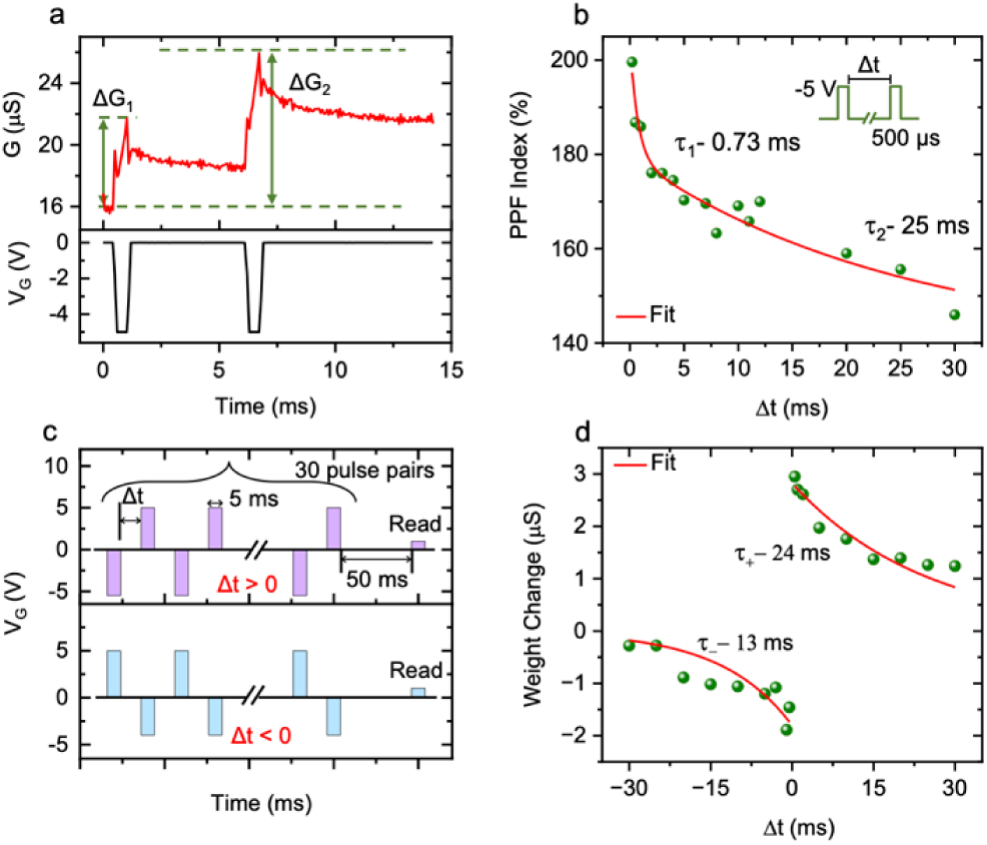
Short-term plasticity demonstrated by the subject Nafion/prGO transistor. (a) Typical PPF behavior obtained under the application of a pair of two identical −*V*_G_ pulses (−5 V), with a width of 500 μs and a 5 ms interval time (Δ*t*). (b) Extracted PPF index plotted as a function of Δ*t*. The red curve is fitted to the experimental results using eq 3. (c) Schematic of pre- and postsynaptic pulse pairs used to mimic Hebbian STDP. (d) Synaptic weight modulation obtained with varied interval time (Δ*t*) between the pre- and postsynaptic pulses. The red curves are fitted to the experimental results by using eq 4.

In the human brain, short-term plasticity (STP) plays a vital role in memory functions such as learning and synaptic weight processing.^34^ Paired-pulse facilitation (PPF), a typical STP feature of a biological synapse, is essential for decoding temporal information in auditory and visual signals.^35^ In this process, a neuron cell undergoes two successive actions, leading to the increased release of neurotransmitters in synaptic vesicles and consequently resulting in an enhanced postsynaptic response. This phenomenon can be replicated effectively in our Nafion/prGO transistor. Figure 5a shows typical PPF behavior, where two identical −*V*_G_ pulses (−5 V) with a 500 μs width were subsequently applied to the gate electrode with a 5 ms interval time (Δ*t*). The conductance level after the second pulse (Δ*G*_2_) became higher than that observed after the first pulse (Δ*G*_1_), relative to the initial value. The PPF index quantifies the postsynaptic response evoked by the second pulse relative to that evoked by the first pulse, which is defined by Δ*G*_2_/Δ*G*_1_ × 100%. Figure 5b shows the PPF index plotted as a function of Δ*t*. The PPF index exhibited the highest value of ∼200% at a minimum Δ*t* of 200 μs and decreased gradually with an increase in Δ*t*. The variation of the PPF index with Δ*t* can be fitted with the standard PPF equation expressed by
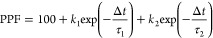
3where *k*_1_*and k*_2_ give the amplitudes of two exponential decay components, while τ_1_ and τ_2_ represent the time constants associated with the respective components. From fitting the PPF curve with eq 3, τ_1_ and τ_2_ were determined to be 0.73 and ∼25 ms, respectively. These time constants are much shorter than those reported for electrolyte-gated transistors^5,10,19,31^ and 2D channel-based transistors.^5,36−38^

Spike-timing-dependent plasticity (STDP), a neurobiological phenomenon observed in biological synapses, holds a pivotal role in Hebbian learning.^39^ To demonstrate STDP behavior with the Nafion/prGO transistor, we input 30 pairs of pre- and postsynaptic spikes with an interval time (Δ*t*). When the presynaptic spike precedes the postsynaptic spike (Δ*t* > 0), the sequence induces potentiation, whereas the reversed sequence (Δ*t* < 0) leads to depression. A schematic of the input pulse pairs used to demonstrate STDP for Δ*t* > 0 and Δ*t* < 0 is presented in Figure 5c. For Δ*t* > 0, the pulse pair consists of a −*V*_G_ pulse (−5.5 V) as the presynaptic spike, followed by a +*V*_G_ pulse (5 V) as the postsynaptic spike. For Δ*t* < 0, the pulse pair consists of a +*V*_G_ pulse (5 V) as the presynaptic spike, followed by a −*V*_G_ pulse (−4 V) as the postsynaptic spike. The pulse width in all cases was fixed at 5 ms. The channel conductance was measured at 50 ms after the last postsynaptic pulse and compared with the initial conductance to calculate the weight change (ξ). Figure 5d plots ξ as a function of Δ*t*, showing that the correlation between the pre- and postsynaptic pulses decreases with increasing Δ*t*. The ξ curves are fitted with the Hebbian STDP learning function given by^40^
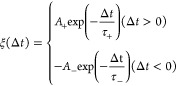
4where *A*^+^ and *A*^–^ represent the maximum conductance change when Δ*t* approaches zero, while τ_+_ and τ_–_ denote the characteristic time constants defining the range of Δ*t* where synaptic strengthening and weakening exhibit significance. From the fitting of ξ curves with eq 4, τ_+_ and τ_–_ were estimated to be ∼24 and ∼13 ms, respectively. These time constants are similar to the time constant of the longer decay component (τ_2_) in the PPF behavior, which indicates that the STDP characteristic is determined by the longer decay of the channel conductance. This time constant range is much shorter than that exhibited by electrolyte-gated transistors^10,19^ and 2D channel-based transistors.^38^

**Figure 6:**
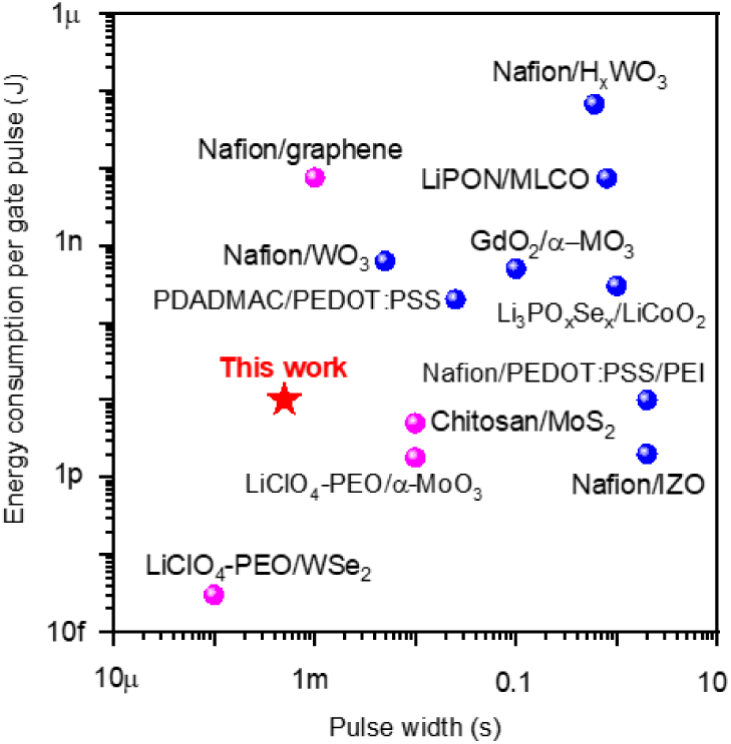
A benchmark plot of the energy consumption per gate pulse vs pulse width, comparing the Nafion/prGO transistor with other reported electrolyte-gated transistors (blue spheres) and 2D channel-based transistors (magenta spheres).

Finally, we discuss the energy efficiency of the Nafion/prGO transistor. From the LTP data with *V*_G_ pulses of −5 V and 1 ms (Figure S11), the average *I*_G_ was estimated to be ∼10 nA, and thus an energy consumption per gate pulse of 10–50 pJ was obtained. Taking a conductance change step of 0.46 μS and a gate electrode area of 200 μm × 100 μm, the energy cost per unit area per unit conductance change was calculated as 1.09–5.43 fJ/(μm^2^ × μS). This value is lower than that exhibited by lithium electrolyte-based transistors^10,34^ and is comparable to Nafion-based transistors with PEDOT:PSS,^32^ WO_3_^12^, and IZO^19^ channels, whose values were obtained by similar calculations. Figure 6 plots the energy consumption of our Nafion/prGO transistor (red pentagram), together with other electrolyte-gated (blue spheres) and 2D channel-based (magenta spheres) transistors, which are operated based on ion movement, as a function of the pulse width. The energy consumptions of electrolyte-gated transistors are distributed between 1 pJ and 0.1 μJ, with pulse widths ranging from 0.1 to 2 s. The time needed to reach equilibrium in the channel is non-negligible due to the finite diffusion time inherent in solids. Therefore, higher energy consumption and longer pulse widths are required. On the other hand, the energy consumptions of 2D channel-based transistors are distributed between 0.01 μJ and 10 fJ, with pulse widths ranging from 100 μs to 10 ms. Such high-speed and energy-efficient operations are attributed to the small volume of the 2D-material channel. Since our transistor possesses 4–5 layers of the prGO channel after electrical reduction, good synaptic characteristics can be realized by taking advantage of the electrolyte-gated and 2D channel-based transistors. A comparison of the performance of our Nafion/prGO transistor with other synaptic transistors is summarized in Table S2.

## Conclusions

In conclusion, we propose a synaptic transistor composed of a prGO channel and a Nafion electrolyte, which is operated by the reduction and oxidation of the prGO channel with protons provided by the Nafion electrolyte. The transistor accommodated shorter write pulses down to 500 μs in width and showcased up to 200 distinct conductance states under gate voltage pulses. Good synaptic functionalities were demonstrated, such as higher weight update linearity, lower energy consumption (10–50 pJ), and a higher on/off ratio of conductance change (∼7). Although our transistor may not show significantly superior characteristics compared to the electrochemical transistors reported so far, ANN simulations based on a two-layer perceptron model, leveraging LTP/LTD characteristics, achieved 90% image recognition accuracy of handwritten digits with voltage pulses and increased to 94% with current pulses due to the improved linear and symmetric conductance change. The transistor also exhibited short-term plasticity, such as PPF and STDP, with time ranges of less than a few tens of milliseconds. These results indicate that the electrolyte-gated prGO transistor has great potential for use in biologically inspired neuromorphic systems, but further investigation is needed to improve the operating characteristics by optimizing the electrolyte materials and device structures.

## Supporting Information Available

Output curves of the pristine Nafion/GO transistor, chemical structures of GO and prGO, Raman spectra measured before and after *V*_DS_ sweeps, variations of Raman spectra at the vicinity of electrodes under voltage sweeps, extinction coefficient vs wavelength of a GO film before and after *V*_DS_ sweeps, photoluminescence spectra of Nafion/GO and Nafion/prGO stacks, LTP/LTD characteristics under application of 200 consecutive +*V*_G_ and −*V*_G_ pulses, retention behavior on a longer time scale in current pulse mode, conductance decay curves after *V*_G_ and *I*_G_ pulses up to 1000 s, humidity effects on LTP/LTD characteristics, normalized conductance vs normalized number of pulses for three different transistors, comparison of nonlinearity and recognition accuracy calculated for three different transistors, time variations of *I*_G_, and channel conductance under *V*_G_ pulses, performance comparison of the subject transistor and other synaptic transistors (PDF)
