# Supplementary material for: Sub-Millisecond and Energy-Efficient Electrochemical Synaptic Transistors with a Partially Reduced Graphene Oxide Channel
Source: ACS Appl Mater Interfaces. 2025 Apr 19;17(17):25674–83. doi: 10.1021/acsami.5c01202 (PMC12051172; doi:10.1021/acsami.5c01202)
Supplement: Supplementary file 1 — am5c01202_si_001.pdf [file am5c01202_si_001.pdf]

Supporting Information

Sub-millisecond and Energy-Efficient Electrochemical Synaptic Transistors with a Partially Reduced Graphene Oxide Channel

Samapika Mallik,* Kazuya Terabe, Tohru Tsuruoka*

Research Center for Materials Nanoarchitectonics (MANA), National Institute for Materials Science, Namiki 1-1, Tsukuba 305-0044, Japan

Corresponding author

Samapika Mallik, Email: ssamapika.mallik@gmail.com

Tohru Tsuruoka, Email: TSURUOKA.Tohru@nims.go.jp

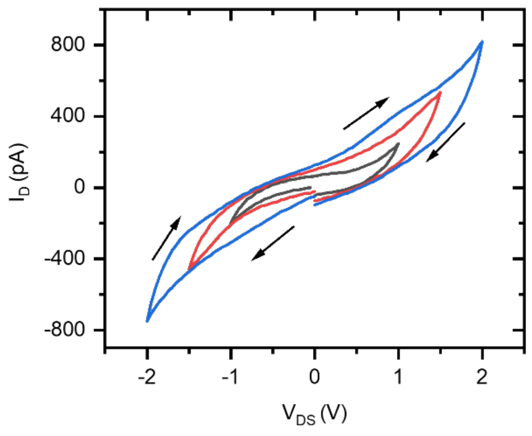

Figure S1. Output curves measured for the pristine Nafion/GO transistor, while increasing the V_{DS} sweep range up to ±2 V.

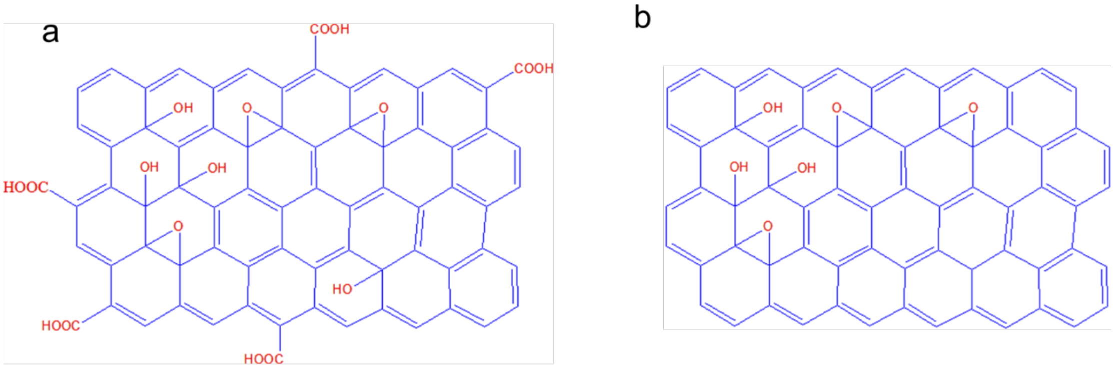

Figure S2. Chemical structure of (a) graphene oxide (GO) and (b) reduced GO.

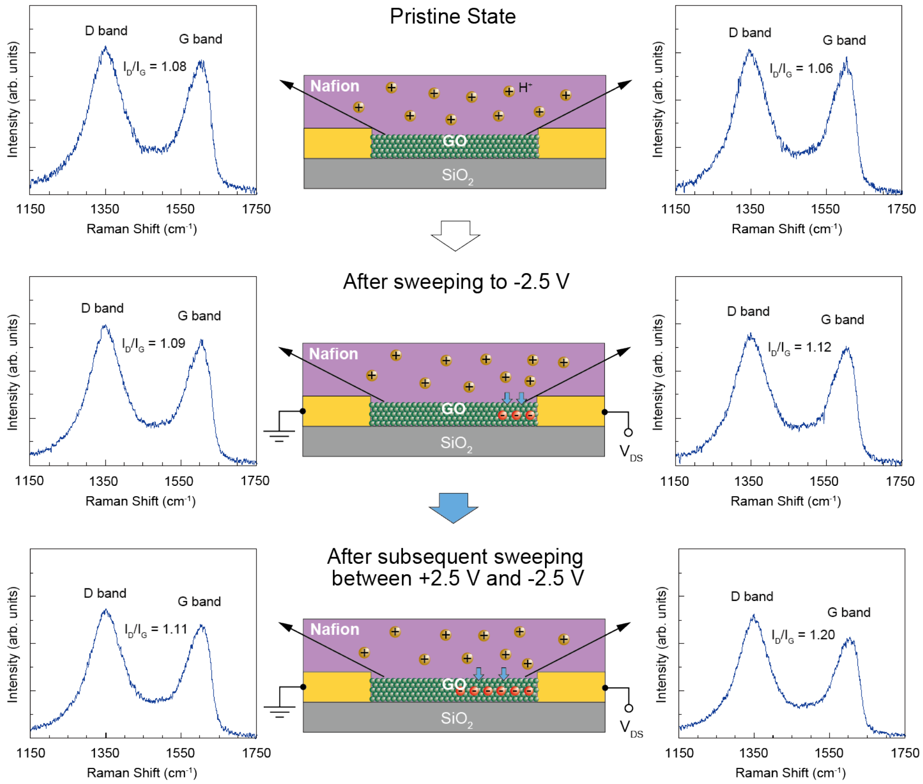

Figure S3. Variations of Raman spectra in the vicinity of both Pt electrodes, measured for the pristine state, after an initial sweep to -2.5 V, and after a subsequent sweep between +2.5 and -2.5 V. In the pristine state, the Raman spectra showed almost the same I_D/I_G ratio at both electrode sides. After the initial sweeping to -2.5 V, the Raman spectrum measured at the negatively biased electrode showed a larger I_D/IG ratio, while the ratio at the grounded electrode was unchanged. After the subsequent sweeping between +2.5 to -2.5 V, the I_D/I_G ratio at the electrode with the initial negative bias further increased, whereas the ratio at the opposite electrode increased only slightly. From this result, it was inferred that GO is reduced from the negatively biased electrode (cathode) side.

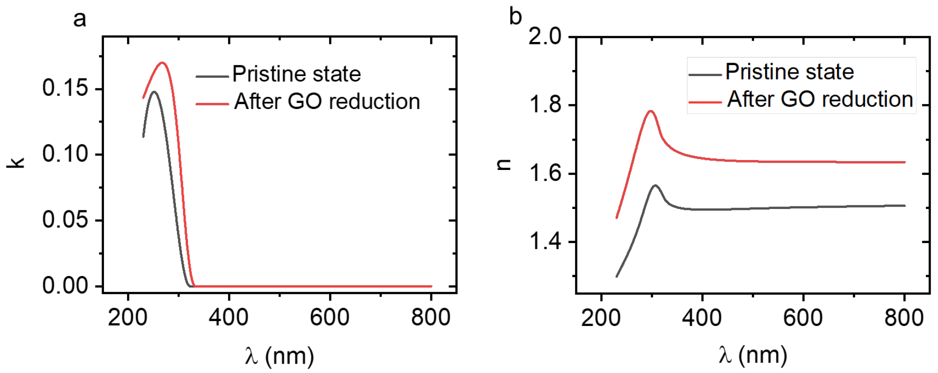

Figure S4. (a) Extinction coefficient (k) versus wavelength (λ) of the GO film before and after V_{DS} sweeps, obtained from the fitting of reflectance spectra with a multi-layer model. (b) Refraction index (n) versus λ.

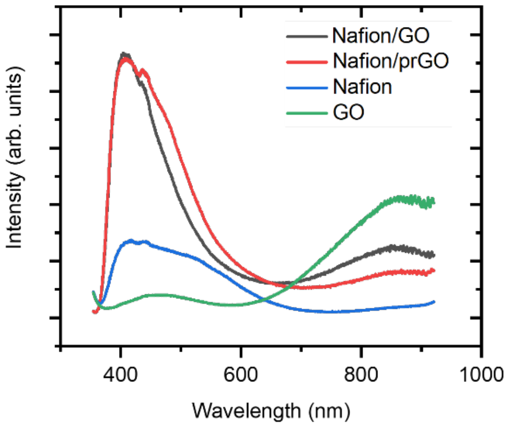

Figure S5. Photoluminescence spectra measured for Nafion film (blue curve), GO film (green curve), and a Nafion/GO stack before (black curve) and after (red curve) electrical reduction of the GO channel by V_{DS} sweeping. All the spectra were measured using a 325-nm line of a He-Cd laser as the excitation source. The PL spectra of both pristine Nafion/GO and Nafion/prGO stacks exhibited an intense PL band at ~400 nm, which band is attributed to an emission from the Nafion overlayer. However, the Nafion/prGO film showed higher PL intensity at ~480 nm but lower PL intensity at ~860 nm, compared to the pristine Nafion/GO film. This variation is consistent with previous works that reported a blue shift of PL emission from a GO film by electrochemical reduction [Chien et al., Angew. Chem. Int. Ed. **2012**, 51, 6662].

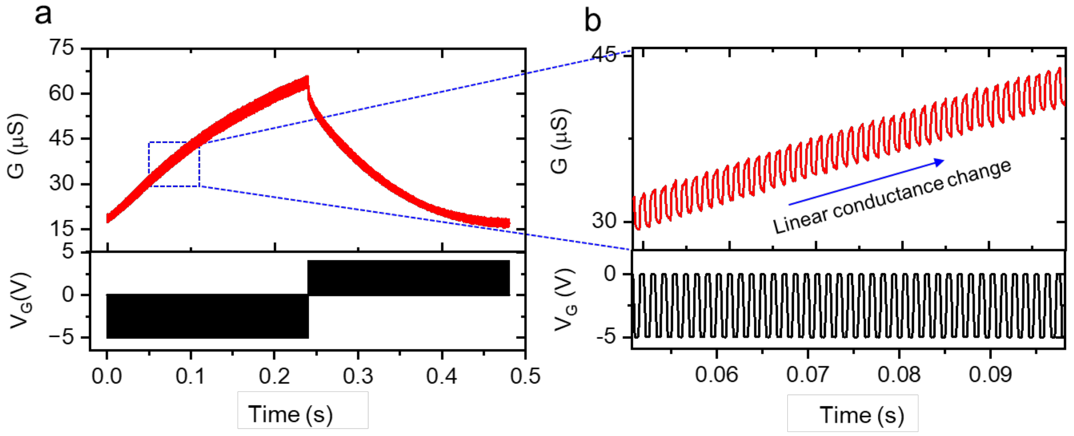

Figure S6. (a) LTP and LTD characteristics of the Nafion/prGO transistor under applications of 200 consecutive positive and negative V_G pulses. (b) Enlarged part of (a) showing a linear conductance change under -V_G pulses.

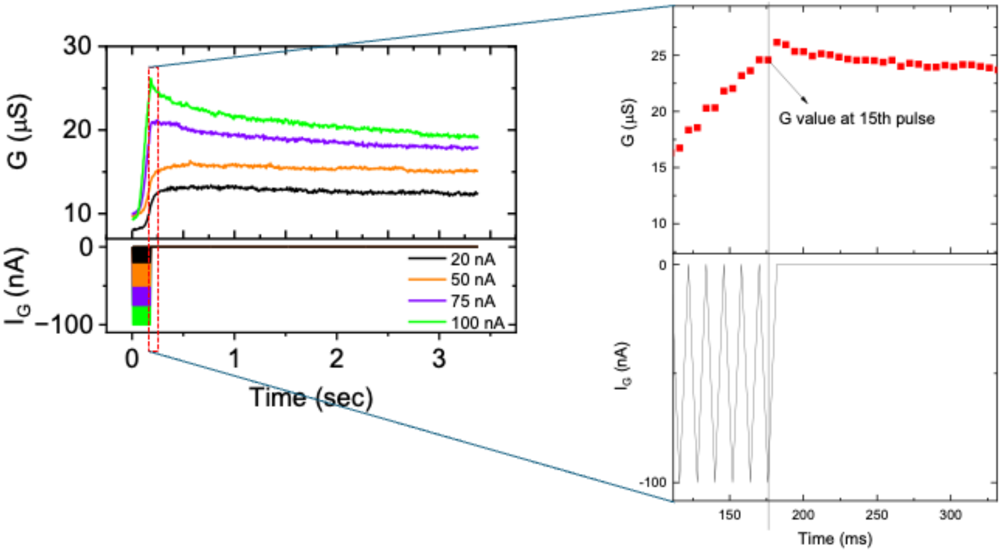

Figure S7. Retention behavior for 15 -I_G pulses (the same as Figure 3d in the manuscript) and the magnified view around the 15th pulse, showing a good retention property for 150 ms.

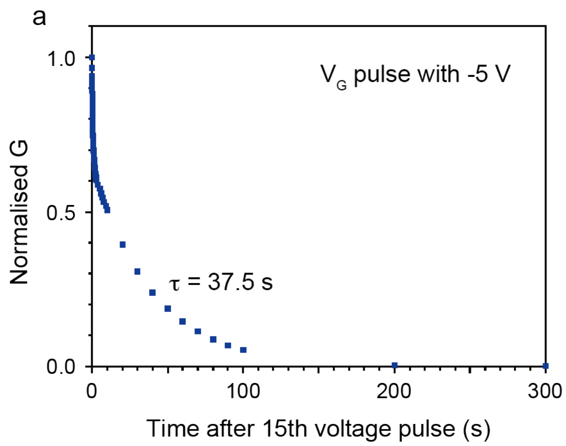

Figure S8. Typical conductance decay curves after application of 15 -V_G pulses (a) and 15 -I_G pulses (b), measured for 1000 s. The retention time τ is defined as the time when the normalized conductance decay below 1/e. τ was estimated to be 37.5 s and ~600 s for the voltage and current pulse modes, respectively. This indicates that current pulses improve the retention characteristics of the Nafion/prGO transistor.

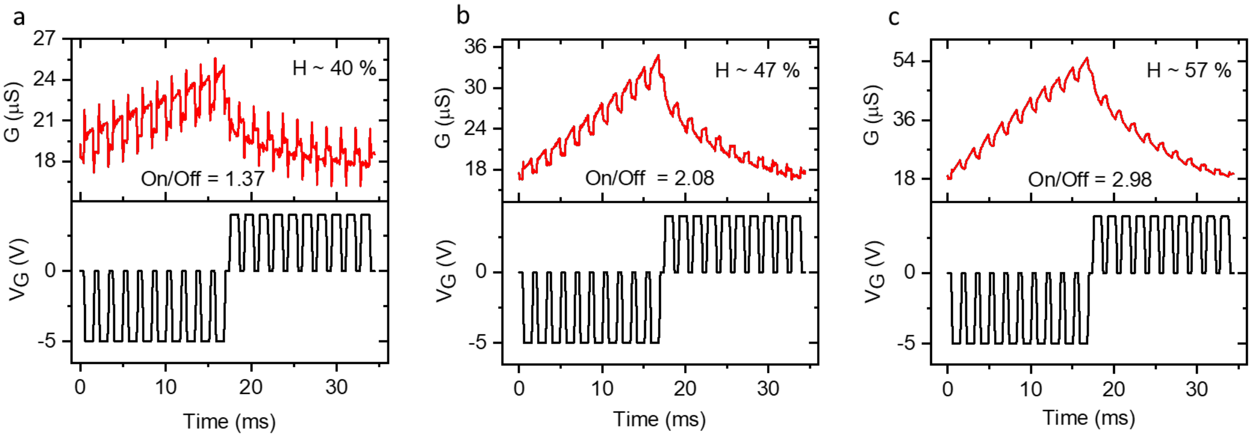

Figure S9. Effects of humidity on the LTP and LTD characteristics of the Nafion/prGO transistor under applications of 10 consecutive positive and negative V_G pulses (5V, 1 ms). Humidity level: (a) ~ 40% (b) ~47% (c) ~57%.

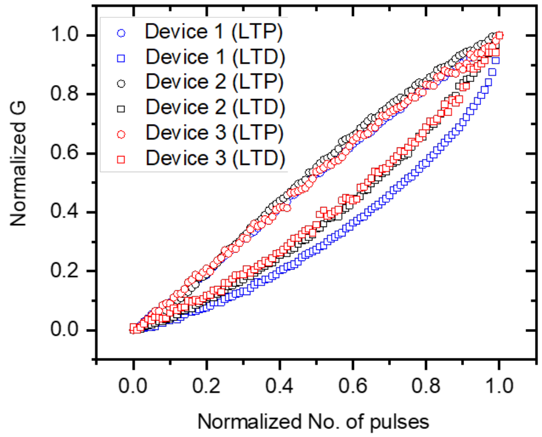

Figure S10. Normalized conductance versus normalized number of pulses, obtained for three different Nafion/prGO transistors in the voltage pulse operation.

Table S1. Comparison of nonlinearity and recognition accuracy calculated for three different Nafion/prGO transistors.

Device	Nonlinearity Factor (N_{LTP})	Nonlinearity Factor (N_{LTD})	Recognition accuracy
1	0.01	-2	89 %
2	0.01	-1.46	91 %
3	0.11	-1.15	90 %

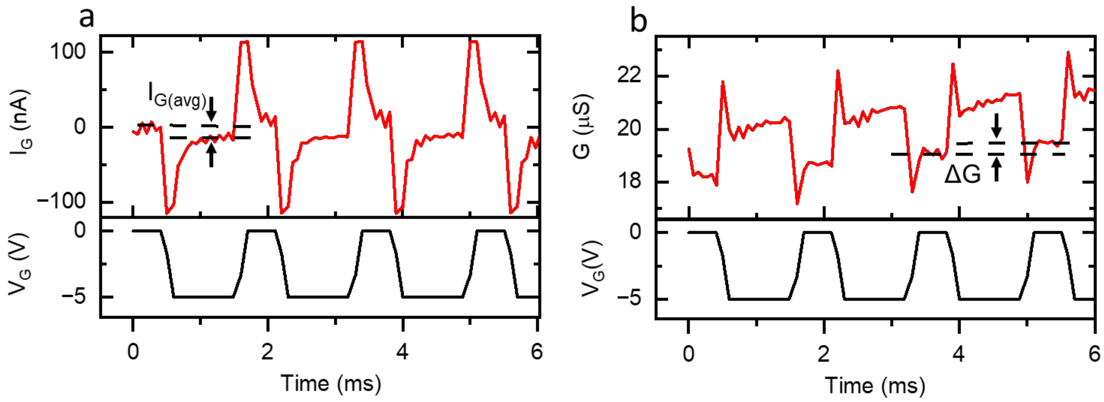

Figure S11. Time variations of (a) I_G and (b) channel conductance G under application of $-V_G$ pulses (-5 V, 1 ms), obtained for the Nafion/prGO transistor. The average gate current and conductance change step are evaluated from the respective waveforms.

Table S2. Performance comparison of our Nafion/prGO transistor device characteristics with other electrolyte-gated transistors and 2D-channel based transistors, which are based on ion movement.

Material Systems	Active Ion	Nonlinearity Factor		Recognition Accuracy (%)		Pulse Width	Energy Consumption	Paper
Electrolyte/Channel		LTP	LTD	Voltage Pulse (%)	Current Pulse (%)			
Nafion/H_XWO_3	Proton	0.8	-3.36	66	88	0.6 s	3.03 µJ/67.5nJ	Neuromorph. Comput. Eng. 3 (2023) 034008.
Nafion/IZO	Proton	1.31	-1.88	84		2 s	2 pJ	ACS Appl. Mater. Interfaces 15 (2023) 19279.
LiPON/MLCO	Li ion	2.2	-2.36	79		0.8 s	7.4 nJ	ACS Appl. Mater. Interfaces 15 (2023) 47184.
GdO_2/α-MO_3	Proton			88		100 ms	0.5 nJ	RSC Adv. 12 (2022) 17706.
MSC/ITO	Proton	-0.47	-0.42	38	94	10 ms		J. Mater. Chem. C 10 (2022) 7241.
K doped AlOx/InO_x	K ion	0.89	-3.54	70		50 ms	2.5 fJ	J. Mater. Chem. C 10 (2022) 3196.
Li_3PO_4/Al_2O_3/WO_x	Li ion	0.69	-0.42	83		1 s		Nanotechnology 32 (2021) 275201.
Na-doped PEO/ZnO	Na ion	0.46	-2.36	91		500 ms		J. Mater. Chem. C 9 (2021) 5396.
Nafion/WO_3	Proton					5 ms	625 pJ	Nat. Commun. 11 (2020) 3134.
$Li_3PO_xSe_x$/$LiCoO_2$	Li ion	1.33	-0.34	91		1 s	300 pJ	Sci. Rep. 9 (2019) 18883.
Li_3PO_4/$LiCoO_2$	Li ion			74			300 pJ	Sci. Rep. 9 (2019) 18883.
PDADMAC/PEDOT:PSS	Proton			88		25 ms	200 pJ	ACS Appl. Mater. Interfaces 11 (2019) 16749.
LiPON/$LiCoO_2$	Li ion				97	2 s		Adv. Mater. 29 (2017) 1604310.
Nafion/PEDOT:PSS/PEI	Proton			97		2 s	10 pJ	Nat. Mater. 16 (2017) 414.
Nafion/graphene	Proton	0.89	-0.76		98	100 µs -1 ms	7.5 nJ (50 aJ/μm^2)	Nat. Commun. 13 (2022) 4386.
Na-diffused SiO_2/MoS_2	Na ion			90		100 ms		Nano Lett. 21 (2021) 10400.
$LiClO_4$-PEO/α-MoO_3	Li ion	0.31	-0.31	87		10 ms	1.8 pJ	Adv. Funct. Mater. 28 (2018) 1804170.
$LiClO_4$-PEO/WSe_2	Li ion					100 µs	30 fJ	Adv. Mater. 2018, 30, 1800195.
Chitosan/MoS_2	Proton					10 ms	5 pJ	J. Mater. Chem. C, 2019, 7, 682.
Nafion/prGO	Proton	0.14	-0.08	90	94	500 µs	10-50 pJ	**This work**

*The energy consumption of some transistors was calculated using the experimental data and device size given in the papers.
